# mHealth Research for Weight Loss, Physical Activity, and Sedentary Behavior: Bibliometric Analysis

**DOI:** 10.2196/35747

**Published:** 2022-06-08

**Authors:** Chieh-Chen Wu, Chih-Wei Huang, Yao-Chin Wang, Md.Mohaimenul Islam, Woon-Man Kung, Yung-Ching Weng, Chun-Hsien Su

**Affiliations:** 1 Department of Exercise and Health Promotion College of Kinesiology and Health Chinese Culture University Taipei Taiwan; 2 Department of Healthcare Information and Management School of Health Technology Ming Chuan University Taipei Taiwan; 3 International Center for Health Information Technology College of Medical Science and Technology Taipei Medical University Taipei Taiwan; 4 Graduate Institute of Injury Prevention and Control Taipei Medical University Taipei Taiwan; 5 AESOP Technology Taipei Taiwan; 6 Graduate Institute of Sport Coaching Science College of Kinesiology and Health Chinese Culture University Taipei Taiwan

**Keywords:** mobile health, weight loss, physical activity, sedentary behavior, bibliometric analysis, mHealth, weight, behavior, research, literature, bibliometric, journal, trend, app

## Abstract

**Background:**

Research into mobile health (mHealth) technologies on weight loss, physical activity, and sedentary behavior has increased substantially over the last decade; however, no research has been published showing the research trend in this field.

**Objective:**

The purpose of this study was to provide a dynamic and longitudinal bibliometric analysis of recent trends of mHealth research for weight loss, physical activity, and sedentary behavior.

**Methods:**

A comprehensive search was conducted through Web of Science to retrieve all existing relevant documents published in English between January 1, 2010, and November 1, 2021. We developed appropriate research questions; based on the proven bibliometric approaches, a search strategy was formulated to screen the title for eligibility. Finally, we conducted bibliometric analyses to explore the growth rate of publications; publication patterns; and the most productive authors, institutions, and countries, and visualized the trends in the field using a keyword co-occurrence network.

**Results:**

The initial search identified 8739 articles, of which 1035 were included in the analyses. Our findings show an exponential growth trend in the number of annual publications of mHealth technology research in these fields. *JMIR mHealth and uHealth* (n=214, 20.67%), *Journal of Medical Internet Research* (n=71, 6.86%), and *BMC Public Health* (n=36, 3.47%) were the top 3 journals, publishing higher numbers of articles. The United States remained the leading contributor in these areas (n=405, 39.13%), followed by Australia (n=154, 14.87%) and England (n=125, 12.07%). Among the universities, the University of Sydney (n=36, 3.47%) contributed the most mHealth technology research in these areas; however, Deakin University (n=25, 2.41%) and the National University of Singapore (n=23, 2.22%) were in the second and third positions, respectively.

**Conclusions:**

Although the number of papers published on mobile technologies for weight loss, physical activity, and sedentary behavior was initially low, there has been an overall increase in these areas in recent years. The findings of the study indicate that mobile apps and technologies have substantial potential to reduce weight, increase physical activity, and change sedentary behavior. Indeed, this study provides a useful overview of the publication trends and valuable guidance on future research directions and perspectives in this rapidly developing field.

## Introduction

Mobile health (mHealth) has emerged as a potential tool to support physicians and patients in many areas [1,2]. Recent evidence shows that mHealth is an easily accessible and cost-effective tool to assist in improving health outcomes [3]. The widespread availability of mobile phones paves the way to conduct advanced research in health care. mHealth-related research is thriving and gaining in popularity [4]. Over the past few years, it has become clear that mHealth technologies (eg, apps or SMS) can help to reduce weight loss, improve physical activity, and change behavior [5-8]. Previous reviews evaluated the effectiveness of mHealth interventions in these three domains [9-12]. These reviews suggest that mHealth interventions appear to be promising for preventive and therapeutic activities. Given the numerous mHealth-related publications, it is important to analyze these research studies to provide an overview of these domains.

Bibliometric analysis is considered a popular and rigorous statistical method for exploring and analyzing a large volume of scientific literature [13]. It can identify the main themes and emerging trends of certain research topics, and detect knowledge in the literature [14]. Bibliometric analyses that summarized the research landscape in various fields have generated valuable insights [14-18], allowing researchers to study specific research areas by analyzing citations, cocitations, geographical distribution, and word frequency, and by providing insightful conclusions [19]. Thus, bibliometric analyses are contributing to monitoring the development and patterns of effective publications. Moreover, bibliometric analyses help researchers, clinicians, and health care policy makers to collect information to understand the particular area of research and their applications, and to promote interdisciplinary collaborations [20].

The aim of this study was to provide a comprehensive picture of mHealth-related research and the direction of future research to benefit the general population, health care policy makers, and researchers.

Based on the research scope and objectives, we developed the following research questions:

- RQ1: What are the basic characteristics of the publications? How many articles on “mobile technologies” for “weight loss, physical activity, and sedentary behavior” have been published between 2010 and 2021?
- RQ2: Who are the most productive authors/coauthors in these areas, and what were the countries of origin?
- RQ3: Which journal published the most? Which organizations mainly contributed to this area?
- RQ4: What are the most frequent cowords (titles/abstract/keywords) associated with these publications?

## Methods

### Search Strategy

We searched for potential publications in Web of Science (WoS) with terms related to mHealth technology, weight loss, physical activity, and sedentary behavior. However, we conducted a comprehensive search in WoS only because it is an extensive database of approximately 10,000 prestigious and high-impact research journals. Moreover, WoS has now been widely used and is the most reliable database for conducting bibliometric analyses [20-22]. WoS contains the following information: title, author, institution, country/region, publication year, citation history, funding source, research types, and keywords [23]. A comprehensive search strategy is presented in Multimedia Appendix 1, Table S1.

### Inclusion and Exclusion Criteria

In our study, all journal articles about mHealth for these three topics were included for screening. The articles were included in the analysis if they (1) were written in the English language; (2) focused on weight loss, physical activity, and/or sedentary behavior; and (3) were involved in mobile technologies. As mHealth is a leading-edge and rapidly updated research area, research or review articles published in peer-reviewed journals, conference proceedings, and early access articles were included. However, letters, editorials, book chapters, and books were excluded from the bibliometric analysis.

### Screening Strategy

Two authors independently screened all the titles and abstracts of retrieved articles and checked the validity of those articles. Any confusion at this stage was resolved by discussing with a third author. Finally, data were collected from selected articles and saved in TXT formats.

### Bibliometric Analysis

We aimed to provide a holistic view of mHealth research on these topics to obtain the knowledge structure, potential authors, research trends, most prolific country and institutions, and research hot spots. Bibliometric analysis was used to show bibliometric maps and the graphical representation of such maps.

#### Growth Rate of Publications

The annual number of publications, annual growth, and average growth rate of publications were calculated in the following ways:



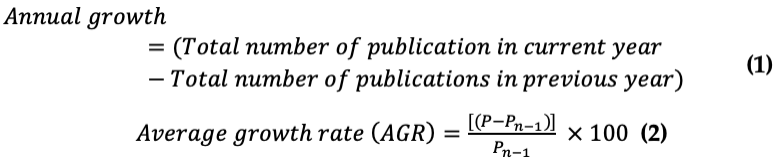



Where *P* is the total number of publications in the current year, and *P_n – 1_* is the total number of publications in the previous year.

#### Publication Patterns

In this study, we also analyzed the publication trends by countries (top 10 most prolific countries), distribution of source journals (top 10 most productive journals), distribution and coauthorship of institutions (top 10 institutions), and distribution of authors (top 10 most productive authors). The rank of the country, journal, institutions, and authors was selected based on the number of publications.

#### Research Hot Spot Tendencies

We developed citation bursts and a timeline map using the VOSviewer software (Centre for Science and Technology Studies, Leiden University). We also constructed and visualized clusters based on publications between 2010 and 2021. However, each cluster was labeled by the keywords provided by the included articles. The top 100 keywords were selected for mapping with their co-occurrence in 5 clusters. A circle with a label illustrates each node in the keyword map: the bigger circles represent higher frequencies. The color of each circle indicates which cluster it belongs to. Finally, the thickness and length of links between nodes show their association strength.

## Results

### Publication Outputs

**Figure 1:**
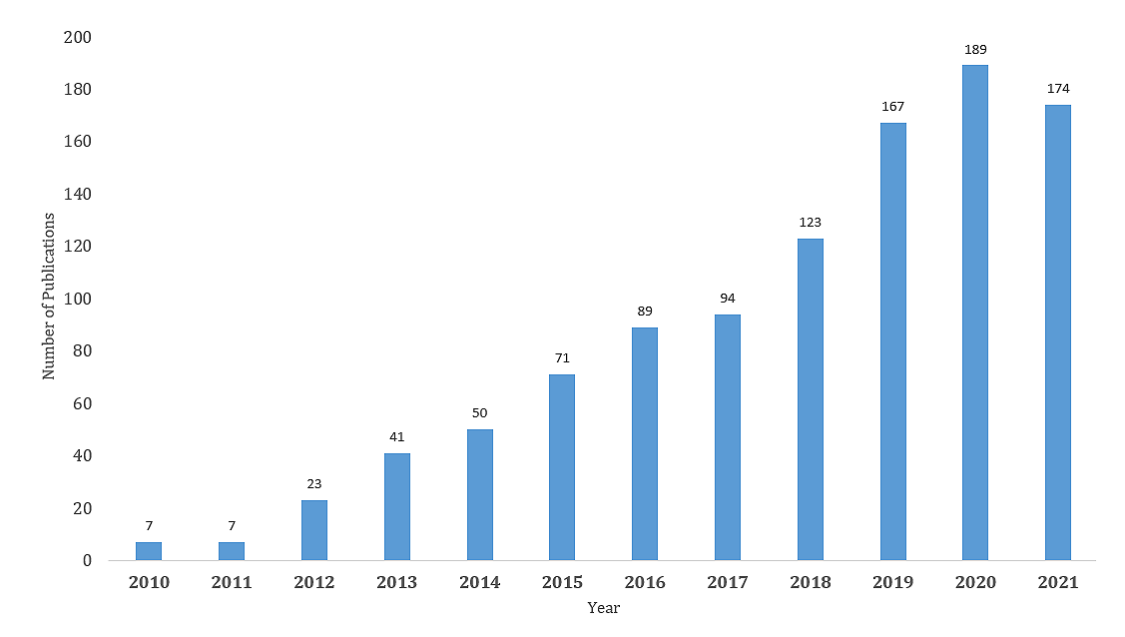
Number of publications on mobile health technologies in these areas between 2010 and 2021.

Based on our comprehensive search on WoS, we identified a total of 8739 articles on mHealth technologies in the three areas (weight loss, physical activity, and/or sedentary behavior). After removing 7704 articles, 1035 articles remained (Table 1). The reasons for the exclusion of studies are given in Multimedia Appendix 1, Figure S1. The number of annual publications on mHealth technologies in these domains increased from 7 articles in 2010 to 173 articles in 2021 (Figure 1). Before 2018, the number of annual articles did not reach 100. The average annual growth rate of articles was a maximum of 228.57% in 2012 and showed a –7.93% decline in 2021.

**Table 1:** The distribution of articles by year between 2010 and 2021.

Year	Publication, n	Annual growth, n	AGR^a^ (%)
2010	7	N/A^b^	N/A
2011	7	0	0.00
2012	23	16	228.57
2013	41	18	78.26
2014	50	9	21.95
2015	71	21	42.00
2016	89	18	25.35
2017	94	5	5.61
2018	123	29	30.85
2019	167	44	35.77
2020	189	22	13.17
2021	174	–15	–7.93

^a^AGR: average growth rate.

^b^N/A: not applicable.

### Distribution of Source Journals

There were a total of 337 journals that published articles on mHealth technologies in these three domains. However, the Canadian *JMIR mHealth and uHealth* was the most productive journal, publishing 214 (20.67%) articles in these three domains (Table 2). *Journal of Medical Internet Research*, *BMC Public Health*, and *International Journal of Environmental Research and Public Health* were in the second, third, and fourth positions, publishing 71, 36, and 30 articles, respectively, on these topics. The top 10 journals published 441 articles, accounting for 42.6% (441/1035) of all publications in these domains.

**Table 2:** Top 10 journals that published articles on mobile health technologies for these three domains, 2010-2021.

Rank	Journal	Country	Categories	Publication (N=1035), n (%)
1	JMIR mHealth and uHealth	Canada	Medical informatics	214 (20.67)
2	Journal of Medical Internet Research	Canada	Medical informatics	71 (6.86)
3	BMC Public Health	England	Public, environmental, and occupational health	36 (3.47)
4	International Journal of Environmental Research and Public Health	Switzerland	Public, environmental, and occupational health	30 (2.89)
5	Translational Behavioral Medicine	Switzerland	Public, environmental, and occupational health	21 (2.02)
6	BMJ Open	England	Medicine (general and internal)	18 (1.73)
7	International Journal of Behavioral Nutrition and Physical Activity	England	Nutrition and dietetics	16 (1.54)
8	PLOS One	United States	Multidisciplinary science	16 (1.54)
9	American Journal of Preventive Medicine	United States	Public, environmental, and occupational health	15 (1.42)
10	Digital Health	England	Public, environmental, and occupational health	14 (1.35)

### Distribution of Coauthorship of Countries/Regions

**Figure 2:**
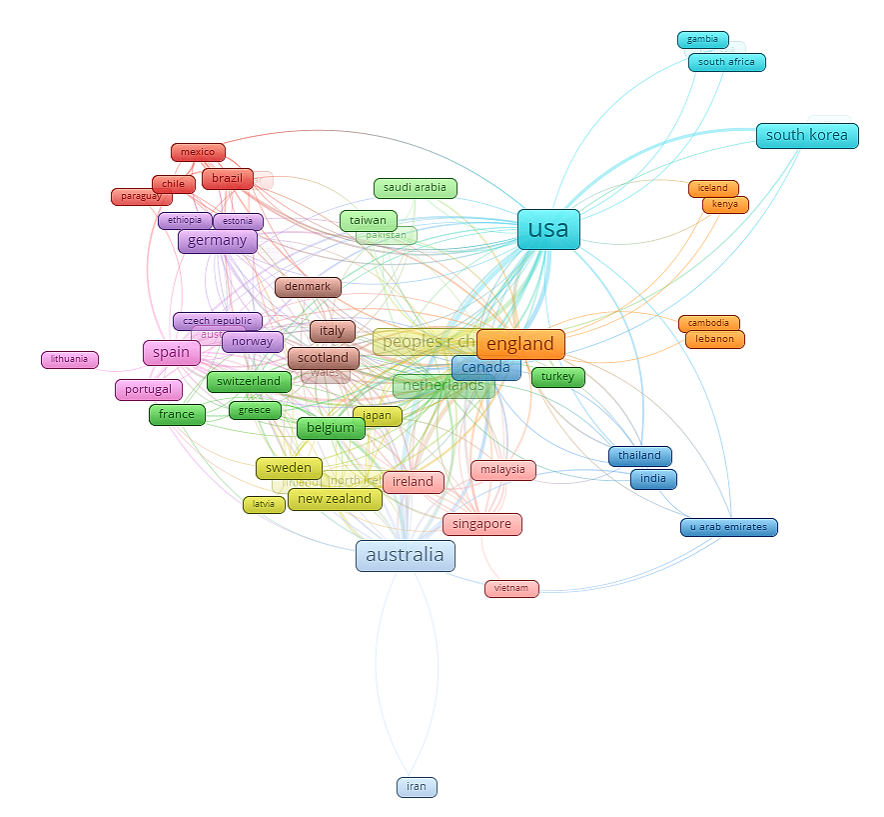
The coauthorship network of countries/regions that published at least one article in these domains, 2010-2021. Peoples R China: People’s Republic of China; U Arab Emirates: United Arab Emirates.

Our study showed that researchers from 73 countries and regions conducted research on these topics and published articles in different international peer-reviewed journals (Figure 2). Of the total 1035 articles, the United States contributed the highest number (n=405, 39.13%), followed by Australia (n=154, 14.87%), England (n=125, 12.07%), China (n=68, 6.57%), Spain (n=60, 5.79%), and Canada (n=52, 5.02%) (Table 3).

**Table 3:** Top 10 countries that published articles on mobile health technologies for these three domains, 2010-2021.

Rank	Country	Publications, n	Citations, n
1	United States	405	12,672
2	Australia	154	4301
3	England	125	4602
4	China	68	819
5	Spain	60	1115
6	Canada	52	876
7	South Korea	49	564
8	Netherlands	43	1383
9	Germany	36	519
10	Ireland	31	571

### Distribution of Coauthorship of Institutions

**Figure 3:**
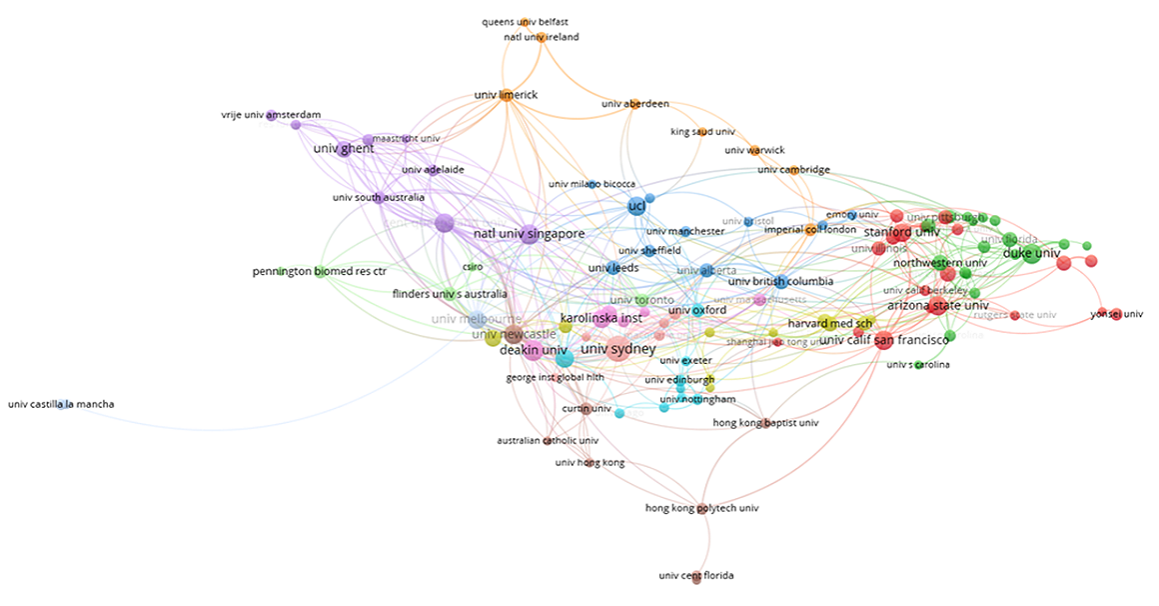
The coauthorship network of institutions that contributed at least 5 articles in these domains, 2010-2021.

According to our study findings, 1494 institutes contributed to at least one study. Table 4 shows the top 10 most productive research institutes that used mHealth technologies in these domains. The University of Sydney (36 articles) ranked first among all research institutions, followed by Deakin University (25 articles), the National University of Singapore (23 articles), and Duke University (22 articles). Figure 3 displays the coauthorship analysis of 117 institutions that published at least 5 articles. It forms a total of 12 clusters (cluster 1, red color, 19 institutions; cluster 2, blue color, 18 institutions; and cluster 12, ash color, 3 institutions), differentiated by various color.

**Table 4:** Top 10 institutions that published papers on mobile health technologies for these three domains, 2010-2021.

Rank	Institution	Country	Publications, n	Citations, n
1	University of Sydney	Australia	36	1009
2	Deakin University	Australia	25	413
3	National University of Singapore	Singapore	23	324
4	Duke University	United States	22	973
5	Central Queensland University	Australia	21	1004
6	University of California, San Francisco	United States	21	569
7	University of Newcastle	Australia	20	754
8	The University of California, Los Angeles	United States	20	1613
9	Arizona State University	United States	20	504
10	University of Auckland	New Zealand	19	882

### Distribution and Coauthorship of Authors

**Figure 4:**
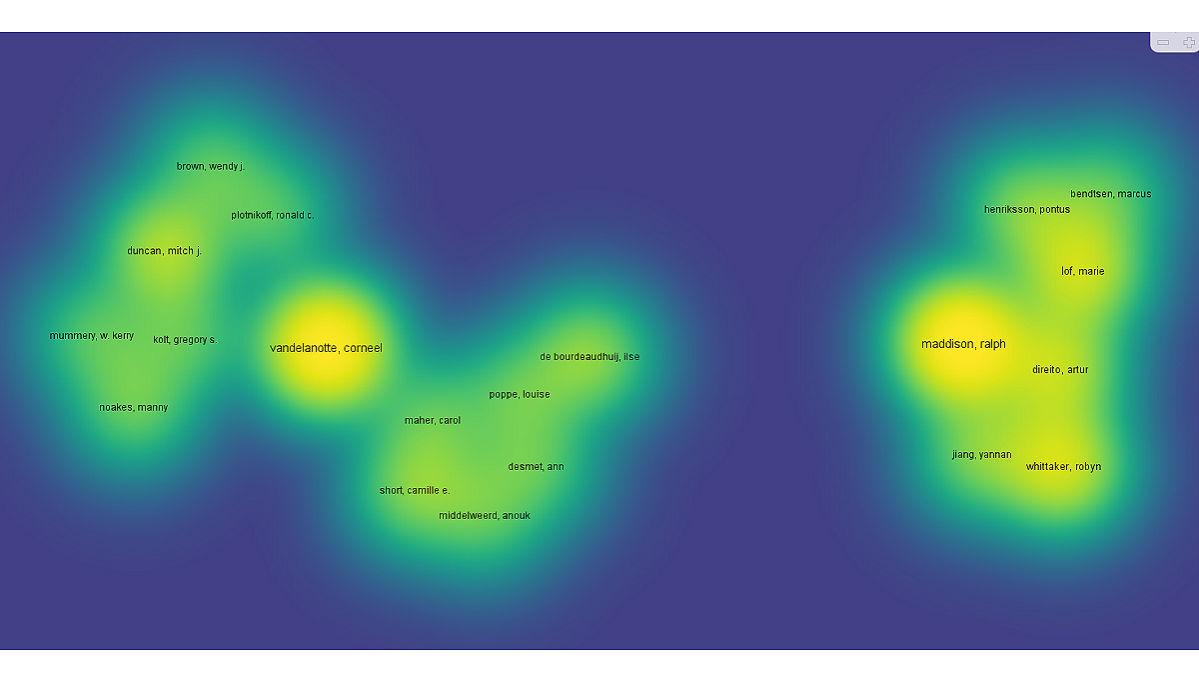
The coauthorship network of authors who contributed research regarding mobile health technologies for these domains, 2010-2021.

Based on our study, 1035 articles were published by 4976 authors with at least one article. Table 5 shows the top 10 most productive authors who conducted and published articles in these domains. Ralph M ranked highest among all authors (23 articles), followed by Corneel V (22 articles), Robyn W (13 articles), and Marie L (12 articles). Our analysis shows that 43 of 4976 authors had published at least 5 articles. The largest set of associated authors consisted of 20 authors in 3 clusters (Figure 4).

**Table 5:** Top 10 authors that published papers on mobile health technologies for these domains, 2010-2021.

Rank	Authors	Publications, n	Citations, n
1	Ralph M	23	775
2	Corneel V	22	1124
3	Robyn W	13	665
4	Marie L	12	68
5	Artur D	11	516
6	Mitch JD	10	551
7	Yoshimi F	9	315
8	Ilse DB	8	85
9	Pontus H	7	29
10	Yannan J	7	290

### Co-occurrence Analysis of Top 100 Keywords

**Figure 5:**
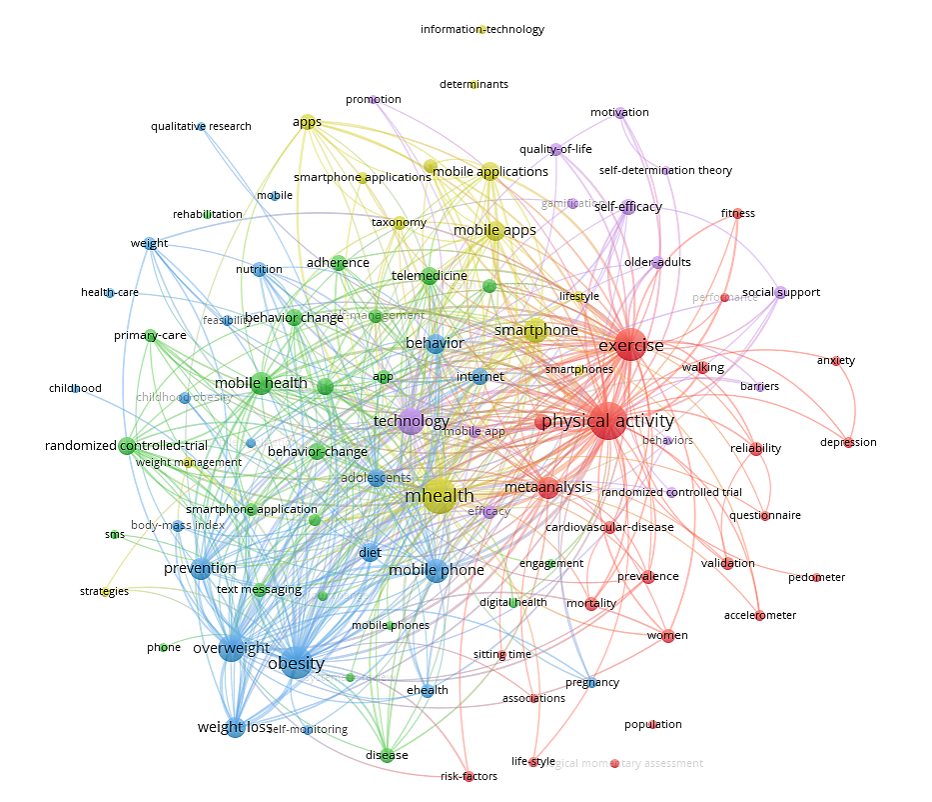
The co-occurrence network of the top 100 keywords in these domains, 2010-2021.

The top 100 keywords were classified into 5 clusters using keyword-clustering analysis (Figure 5). The five most common keywords for mHealth technologies were physical activity (n=282), mHealth (n=260), exercise (n=220), obesity (n=220), and health (n=220). The 5 clusters are represented by color: red (cluster 1), green (cluster 2), blue (cluster 3), yellow (cluster 4), and violet (cluster 5). The node labels are the keywords, and the node size depends on the number of keyword co-occurrences. The links connecting two nodes show a co-occurrence relationship between the keywords.

## Discussion

### Main Findings

The use of mHealth technologies has been rapidly increasing. mHealth technologies are virtually ubiquitous and provide great opportunities to deliver health care management and health promotion to people in low-resource settings and with limited access to care [24-26]. It also facilitates the tracking of activity, behavior, and weight, allowing for real-time recording, feedback, and accountability [27]. Previous studies have shown that mHealth technologies support a reduction in obesity, improving physical activity and reducing the risk of developing life-threatening complications [28-30].

This study explored the bibliometric analyses of mHealth intervention research in weight loss, physical activity, and sedentary behavior. By analyzing the results of the data set regarding the publication pattern on research related to mHealth technology with these areas, we found a rapid increase in the growth of interest in this subject in the last decade. The trend substantially increased in the most recent years, from 2018 to 2021. This increased trend of publication could reflect the improvements, functionality, and developments of mHealth research in parallel with comparison to other areas of use. Therefore, mHealth offers patients many high-quality choices in the self-management of their chronic diseases, provides new opportunities and convenience, and addresses potential benefits. However, an effective cooperation among clinical experts, mHealth technology developers, and providers must achieve the ultimate goal and objectives. The primary objective of this study was to highlight the most frequent subject categories, along with popular keywords and terms. These aspects reflected recent research trends and influential articles, authors, and collaboration networks among researchers and institutions. Our findings show that the United States was the most substantial contributor, and 4 of the 10 most productive institutions were from the United States. The *Journal of Medical Internet Research* was also the leading journal publishing research in these three areas as well as a bibliometric study that examined the overall mHealth literature [15].

### Global Trends in mHealth Research

The emergence of mHealth technologies has opened up new opportunities to improve patient care in many ways. The obstacles of remote locations and accessibility to a wide range of populations, especially in areas with minimum medical resources, have been reported. The publication growth trends on these domains between 2010 and 2021 had increased exponentially. This suggests that the acceptability of mHealth research on these domains has been increased, and research into mHealth progressed relatively higher. Since the number of mHealth users is gradually increasing, more and more researchers are focusing on these domains, while the number of mHealth-related publications are showing increased trends [31,32]. The number of research papers published in these areas since 2010 was more than 1000. For journal sources, the top 3 journals publishing mHealth in these domains belong to the area of medical informatics and public health. As mHealth technologies evaluated weight management, increasing physical activity, and changing sedentary behavior, the authors preferred top-ranking and reputed journals in medical informatics and public-related journals. *JMIR mHealth and uHealth*, the *Journal of Medical Internet Research*, and *BMC Public Health* are the most popular open-access journals and the most popular outlet for researchers in these fields. Moreover, journals that publish open access obtain a higher number of citations than non–open access journals [33,34].

### International Trends

The findings of our study show that the United States remained the most productive country in these areas. The number of annual publications in the United States had steadily increased. This is followed by Australia, England, China, Spain, and Canada, which were experiencing the rapid growth of research in these areas using mHealth technologies. The vast majority (approximately 97%) of American adults own a mobile phone [35,36], which opens up the opportunity to conduct research that is particularly relevant to the management of chronic diseases [37,38]. A previous study reported that more than 40% of US adults have two or more chronic diseases [39] and 71% of all US health care spending is for chronic diseases [40]. The prevalence of obesity in the United States, England, and Australia are 37.7% [41], 28% [42], and 26% [43], respectively. Obesity and sedentary behavior are the major risk factors for several chronic conditions such as dyslipidemia [44], cardiovascular disease [45], and cancer [46]. These conditions are also associated with detrimental psychological, social, and economic consequences [47]. Therefore, mHealth technologies have become more attractive for conducting research in these domains. The five most productive countries had potential research collaboration in these domains. A coauthorship network always reflects the collaborative relationship among researchers and provides possible opportunities for other researchers to collaborate. The most productive European countries (England, Netherlands, Spain, Ireland, and Norway) had close collaboration. However, they had a strong collaboration with the United States and Australia. Moreover, Asian countries such as China, Korea, and India were the most productive countries and established strong research collaboration with the United States, England, Netherland, Canada, and Australia. However, international research collaboration always depends on several key factors such as international relations, geography, and political and economic alliances [25].

### Limitations

Even though our study provided a comprehensive picture of mHealth research on weight loss, physical activity, and sedentary behavior, there are still several limitations that need to be addressed. First, we collected data from a single database. Although, WoS is an extensive database that offers a wide variety of publications needed for the comprehensive analysis of any topic [25,48]. However, future studies might include other popular databases such as Scopus or PubMed to include more potential studies. Second, we included only studies published in English; however, we might have missed some publications due to this language restriction. Third, we did not consider gray articles and published material such as meeting abstracts, letters, and editorials. Finally, we were unable to conduct individual analyses of weight loss, physical activity, and sedentary behavior due to the limited number of studies.

### Conclusion

We aimed to present a clear picture of mHealth-related research in weight loss, physical activity, and sedentary behavior. Likely, the findings of this study show that the growth rate of mHealth research regarding these three domains has been rapid in the past decade and the annual growth rate is continuously growing; high-income countries like the United States, England, and Australia are the main force behind mHealth-related research on these topics; and medical informatics journals such as *JMIR mHealth and uHealth* and the *Journal of Medical Internet Research* are the top contributors to this topic based on the amount of articles published. Since mHealth research into health care, including these three topics, is accelerating rapidly, these findings can assist researchers and health care policy makers in taking proper directions in future research.

## Appendix

Multimedia Appendix 1 Supplementary table and figure.

## Abbreviations

mHealth: mobile health
WoS: Web of Science

## References

[1] Islam MM, Poly TN, Walther BA, Jack Li YC (2020). Use of mobile phone app interventions to promote weight loss: meta-analysis. JMIR Mhealth Uhealth.

[2] Silva BM, Rodrigues JJ, de la Torre Díez I, López-Coronado M, Saleem K (2015). Mobile-health: a review of current state in 2015. J Biomed Inform.

[3] Kahn JG, Yang JS, Kahn JS (2010). 'Mobile' health needs and opportunities in developing countries. Health Aff (Millwood).

[4] Gilmore LA, Klempel MC, Martin CK, Myers CA, Burton JH, Sutton EF, Redman LM (2017). Personalized mobile health intervention for health and weight loss in postpartum women receiving women, infants, and children benefit: a randomized controlled pilot study. J Womens Health (Larchmt).

[5] Jatobá LC, Grossmann U, Kunze C, Ottenbacher J, Stork W (2008). Context-aware mobile health monitoring: evaluation of different pattern recognition methods for classification of physical activity. Annu Int Conf IEEE Eng Med Biol Soc.

[6] Lee AM, Chavez S, Bian J, Thompson LA, Gurka MJ, Williamson VG, Modave F (2019). Efficacy and effectiveness of mobile health technologies for facilitating physical activity in adolescents: scoping review. JMIR Mhealth Uhealth.

[7] Buckingham SA, Williams AJ, Morrissey K, Price L, Harrison J (2019). Mobile health interventions to promote physical activity and reduce sedentary behaviour in the workplace: a systematic review. Digit Health.

[8] Shaw RJ, Bosworth HB, Silva SS, Lipkus IM, Davis LL, Sha RS, Johnson CM (2013). Mobile health messages help sustain recent weight loss. Am J Med.

[9] Jung J, Cho I (2022). Promoting physical activity and weight loss with mHealth interventions among workers: systematic review and meta-analysis of randomized controlled trials. JMIR Mhealth Uhealth.

[10] Flores Mateo G, Granado-Font E, Ferré-Grau C, Montaña-Carreras X (2015). Mobile phone apps to promote weight loss and increase physical activity: a systematic review and meta-analysis. J Med Internet Res.

[11] Yerrakalva D, Yerrakalva D, Hajna S, Griffin S (2019). Effects of mobile health app interventions on sedentary time, physical activity, and fitness in older adults: systematic review and meta-analysis. J Med Internet Res.

[12] Compernolle S, DeSmet A, Poppe L, Crombez G, De Bourdeaudhuij I, Cardon G, van der Ploeg HP, Van Dyck D (2019). Effectiveness of interventions using self-monitoring to reduce sedentary behavior in adults: a systematic review and meta-analysis. Int J Behav Nutr Phys Act.

[13] Donthu N, Kumar S, Mukherjee D, Pandey N, Lim WM (2021). How to conduct a bibliometric analysis: an overview and guidelines. J Business Res.

[14] Guo Y, Hao Z, Zhao S, Gong J, Yang F (2020). Artificial intelligence in health care: bibliometric analysis. J Med Internet Res.

[15] Sweileh WM, Al-Jabi SW, AbuTaha AS, Zyoud SH, Anayah FMA, Sawalha AF (2017). Bibliometric analysis of worldwide scientific literature in mobile - health: 2006-2016. BMC Med Inform Decis Mak.

[16] López-Robles JR, Otegi-Olaso JR, Porto-Gómez I (2018). Bibliometric analysis of worldwide scientific literature in Project Management Techniques and Tools over the past 50 years: 1967-2017.

[17] Adunlin G, Diaby V, Xiao H (2015). Application of multicriteria decision analysis in health care: a systematic review and bibliometric analysis. Health Expect.

[18] Diaby V, Campbell K, Goeree R (2013). Multi-criteria decision analysis (MCDA) in health care: a bibliometric analysis. Operations Res Health Care.

[19] Liao H, Tang M, Luo L, Li C, Chiclana F, Zeng X (2018). A bibliometric analysis and visualization of medical big data research. Sustainability.

[20] Kan W, Chou W, Chien T, Yeh Y, Chou P (2020). The most-cited authors who published papers in JMIR mHealth and uHealth using the authorship-weighted scheme: bibliometric analysis. JMIR Mhealth Uhealth.

[21] Ahmadvand A, Kavanagh D, Clark M, Drennan J, Nissen L (2019). Trends and visibility of "Digital Health" as a keyword in articles by JMIR Publications in the new millennium: bibliographic-bibliometric analysis. J Med Internet Res.

[22] Peng C, He M, Cutrona SL, Kiefe CI, Liu F, Wang Z (2020). Theme trends and knowledge structure on mobile health apps: bibliometric analysis. JMIR Mhealth Uhealth.

[23] Islam M, Poly T, Alsinglawi B, Lin LF, Chien SC, Liu JC, Jian WS (2021). Application of artificial intelligence in COVID-19 pandemic: bibliometric analysis. Healthcare (Basel).

[24] Quintiliani LM, Mann DM, Puputti M, Quinn E, Bowen DJ (2016). Pilot and feasibility test of a mobile health-supported behavioral counseling intervention for weight management among breast cancer survivors. JMIR Cancer.

[25] Cao J, Lim Y, Sengoku S, Guo X, Kodama K (2021). Exploring the shift in international trends in mobile health research from 2000 to 2020: bibliometric analysis. JMIR Mhealth Uhealth.

[26] Chen M, Wu T, Lv M, Chen C, Fang Z, Zeng Z, Qian J, Jiang S, Chen W, Zhang J (2021). Efficacy of mobile health in patients with low back pain: systematic review and meta-analysis of randomized controlled trials. JMIR Mhealth Uhealth.

[27] Nicklas JM, Leiferman JA, Lockhart S, Daly KM, Bull SS, Barbour LA (2020). Development and modification of a mobile health program to promote postpartum weight loss in women at elevated risk for cardiometabolic disease: single-arm pilot study. JMIR Form Res.

[28] Stork MJ, Bell EG, Jung ME (2021). Examining the impact of a mobile health app on functional movement and physical fitness: pilot pragmatic randomized controlled trial. JMIR Mhealth Uhealth.

[29] Qian J, Wu T, Lv M, Fang Z, Chen M, Zeng Z, Jiang S, Chen W, Zhang J (2021). The value of mobile health in improving breastfeeding outcomes among perinatal or postpartum women: systematic review and meta-analysis of randomized controlled trials. JMIR Mhealth Uhealth.

[30] Kim M, Kim C, Kim E, Choi M (2021). Effectiveness of mobile health-based exercise interventions for patients with peripheral artery disease: systematic review and meta-analysis. JMIR Mhealth Uhealth.

[31] Robbins R, Krebs P, Jagannathan R, Jean-Louis G, Duncan DT (2017). Health app use among US mobile phone users: analysis of trends by chronic disease status. JMIR Mhealth Uhealth.

[32] Luxton DD, McCann RA, Bush NE, Mishkind MC, Reger GM (2011). mHealth for mental health: integrating smartphone technology in behavioral healthcare. Professional Psychol Res Pract.

[33] Ottaviani J (2016). The post-embargo open access citation advantage: it exists (probably), its modest (usually), and the rich get richer (of course). PLoS One.

[34] Eysenbach G (2006). Citation advantage of open access articles. PLoS Biol.

[35] Iliescu R, Kumaravel A, Smurawska L, Torous J, Keshavan M (2021). Smartphone ownership and use of mental health applications by psychiatric inpatients. Psychiatry Res.

[36] Gmunder KN, Ruiz JW, Franceschi D, Suarez MM (2021). Demographics associated with US healthcare disparities are exacerbated by the telemedicine surge during the COVID-19 pandemic. J Telemed Telecare.

[37] Moore SL, Fischer HH, Steele AW, Joshua Durfee M, Ginosar D, Rice-Peterson C, Berschling JD, Davidson AJ (2014). A mobile health infrastructure to support underserved patients with chronic disease. Healthc (Amst).

[38] Yu SWY, Hill C, Ricks ML, Bennet J, Oriol NE (2017). The scope and impact of mobile health clinics in the United States: a literature review. Int J Equity Health.

[39] Buttorff C, Ruder T, Bauman M (2017). Multiple Chronic Conditions in the United States.

[40] Leroy L, Bayliss E, Domino M, Miller BF, Rust G, Gerteis J, Miller T, AHRQ MCC Research Network (2014). The Agency for Healthcare Research and Quality Multiple Chronic Conditions Research Network: overview of research contributions and future priorities. Med Care.

[41] Flegal KM, Kruszon-Moran D, Carroll MD, Fryar CD, Ogden CL (2016). Trends in obesity among adults in the United States, 2005 to 2014. JAMA.

[42] Gurka MJ, Filipp SL, DeBoer MD (2018). Geographical variation in the prevalence of obesity, metabolic syndrome, and diabetes among US adults. Nutr Diabetes.

[43] Keramat SA, Alam K, Al-Hanawi MK, Gow J, Biddle SJH, Hashmi R (2021). Trends﻿ in the prevalence of adult overweight and obesity in Australia, and its association with geographic remoteness. Sci Rep.

[44] Bays HE, Toth PP, Kris-Etherton PM, Abate N, Aronne LJ, Brown WV, Gonzalez-Campoy JM, Jones SR, Kumar R, La Forge R, Samuel VT (2013). Obesity, adiposity, and dyslipidemia: a consensus statement from the National Lipid Association. J Clin Lipidol.

[45] Piché ME, Poirier P, Lemieux I, Després JP (2018). Overview of epidemiology and contribution of obesity and body fat distribution to cardiovascular disease: an update. Prog Cardiovasc Dis.

[46] De Pergola G, Silvestris F (2013). Obesity as a major risk factor for cancer. J Obes.

[47] Apovian C (2016). Obesity: definition, comorbidities, causes, and burden. Am J Manag Care.

[48] Ghanbari M, Behzadifar M, Doshmangir L, Martini M, Bakhtiari A, Alikhani M, Bragazzi NL (2021). Mapping research trends of universal health coverage from 1990 to 2019: bibliometric analysis. JMIR Public Health Surveill.

