# Supplementary material for: mHealth Research for Weight Loss, Physical Activity, and Sedentary Behavior: Bibliometric Analysis
Source: J Med Internet Res. 2022 Jun 8;24(6):e35747. doi: 10.2196/35747 (PMC9218882; doi:10.2196/35747)
Supplement: Multimedia Appendix 1 [file jmir_v24i6e35747_app1.docx]

**Supplementary table S1:** Search strategy:

**Step 1**: Search 1:“mhealth” OR “mobile health” OR “cell-phone” OR “cell phone” OR “mobile phone” OR “mobile-phone” OR “smartphone” OR “smart phone” OR “iphone” OR “mobile apps” OR “Apps” OR “mobile applicat*” OR “text-messaging” OR “mobile device” OR “mobile app*” OR “short message service” OR “sms”

**Step 2**: Search 2: "physical activit*" OR "motor activit*" OR "outdoor activit*" OR exercis* OR walk* OR "active transport*" OR "active living" OR "leisure activit*" OR fitness OR acceleromet* OR pedomet* OR sedentar* OR "sitting time" * OR "weight loss" OR "weight maintenance" OR "maintaining weight" OR "weight gain" OR "weight management" OR “overweight” OR obes* OR lifestyle* OR "behavio* change*" OR "behavio*intervent*")

**Step 3:** Search 1 and Search 2

Supplementary figure S1:

Articles identified through initial search

N = 8,739

Excluded 229 articles due to meeting abstract: 203, editorial: 15, letter: 7, correction: 4

N =

Articles excluded based on title

N = 7,475

Articles included in this study

N = 1,035

Articles after initial screening

N = 1,264
